# Supplementary material for: Flavonoid-Modifying Capabilities of the Human Gut Microbiome—An In Silico Study
Source: Nutrients. 2021 Aug 3;13(8):2688. doi: 10.3390/nu13082688 (PMC8398226; doi:10.3390/nu13082688)
Supplement: Supplementary file 1 [file nutrients-13-02688-s001.zip › Supplementary material.pdf]

Supplementary information to

Flavonoid-modifying capabilities of the gut microbiome – an *in silico* study

Tobias Goris[1], Rafael Cuadrat[2], Annett Braune[1]

[1]Department of Molecular Toxicology, Research Group Intestinal Microbiology, German Institute of Human Nutrition Potsdam-Rehbruecke, Arthur-Scheunert-Allee 114-116, 14558 Nuthetal, Germany

[2]Department of Epidemiology, German Institute of Human Nutrition Potsdam-Rehbruecke, Arthur-Scheunert-Allee 114-116, 14558 Nuthetal, Germany

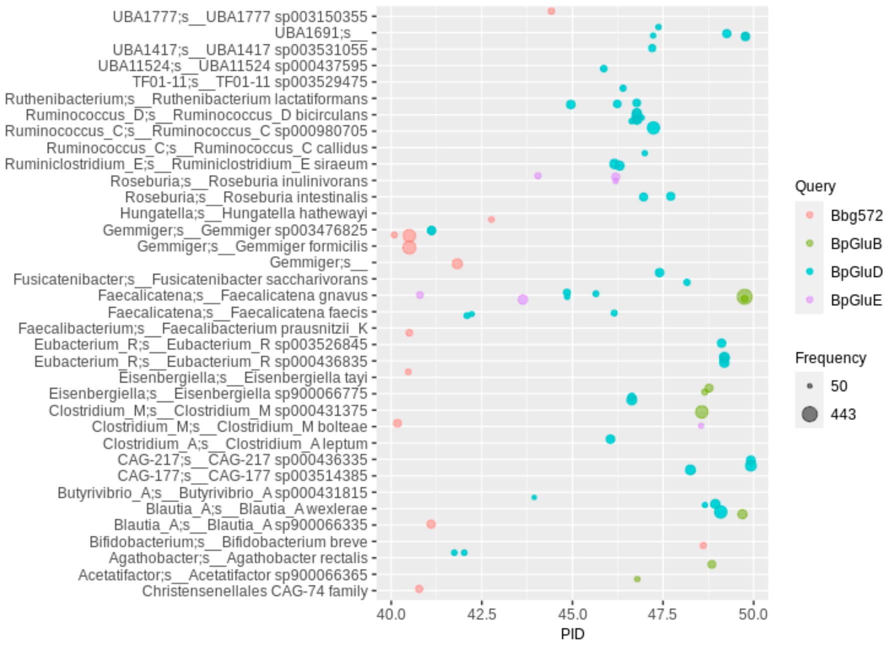

Supplementary Figure S1: Hits of human gut MAGs to flavonoid *O*-deglycosidases. A PID threshold of 40 to 50 was chosen to the queries shown in the color code (for abbreviations and details see Table 1). Hits were filtered for at least 50 occurrences, so that each bubble represents a number of redundant sequences ranging from 50 to 443.

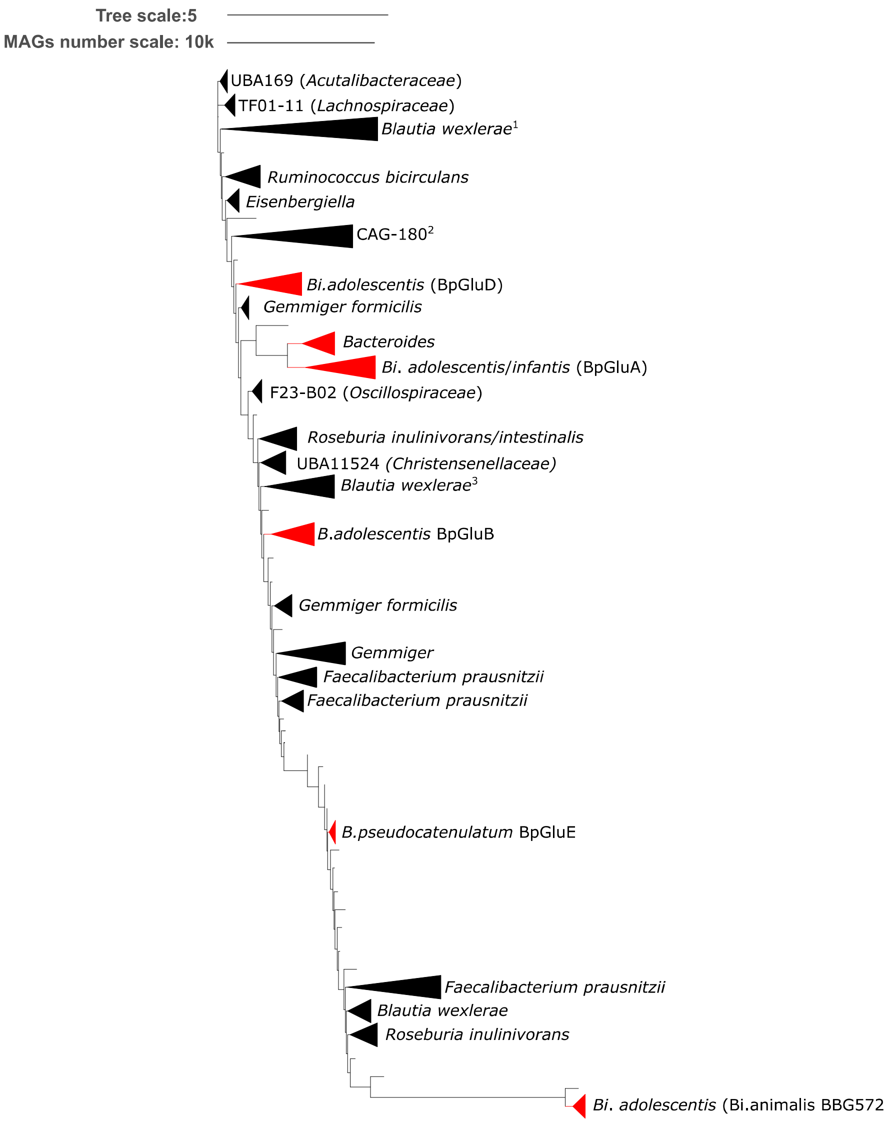

Supplementary Figure S2: Phylogenetic tree of *O*-deglycosidase sequences. Most abundant species genomes are indicated besides the triangles. Colored in red are nodes containing the six reference sequences of *Bifidobacteria* and *Bacteroides* species (in brackets, for abbreviations see Table 1). Only nodes with an overall number of at least 500 genomes (including isolate genomes) were included as triangles, the lengths of the triangles represent the number of genomes as indicated by the scale. [1]: Heterogenous group, also *Ruminoclostridium siraeum* and *Ruminococcus* species highly abundant. [2]: Heterogenous group, also high abundance of CAG-177 and *Eubacterium* genomes. [3]: Heterogenous group, also high abundance of *Faecalicatena gnavus* genomes.

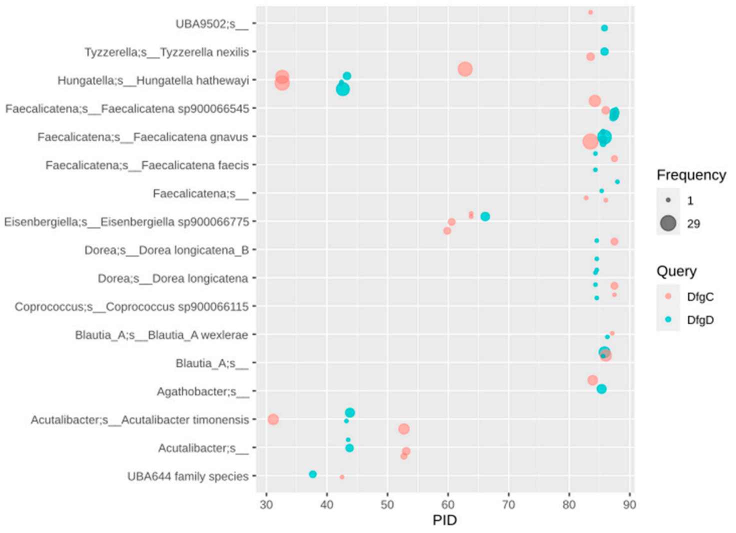

Supplementary Figure S3: Hits to sequences of the flavonoid *O*-deglycosidase DfgCD from *E. ramulus*. Hits were filtered for MAGs in which both genes were co-occurring.

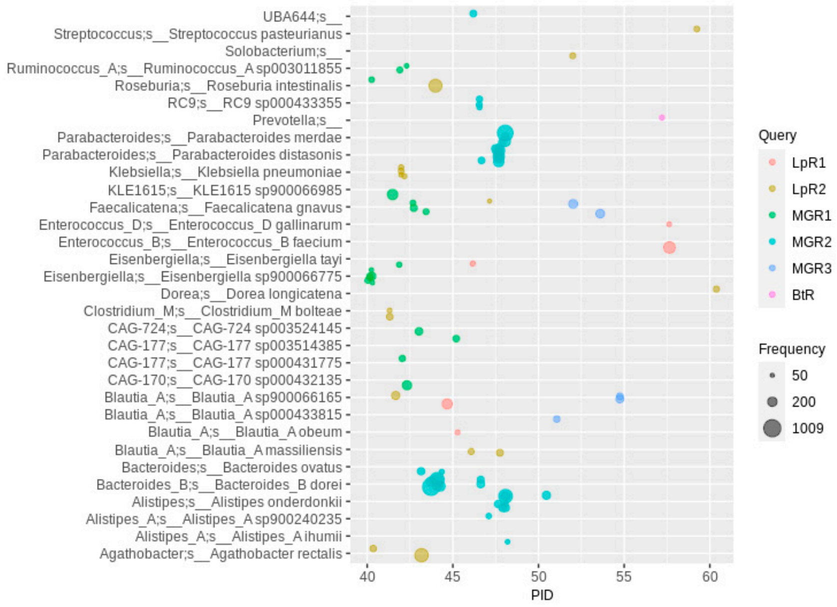

Supplementary Figure S4: Hits with 40 to 65 PID to flavonoid rhamnosidase sequences. Filtered for at least 50 occurrences per redundant sequence so that each bubble represents 50 to 1009 sequences.

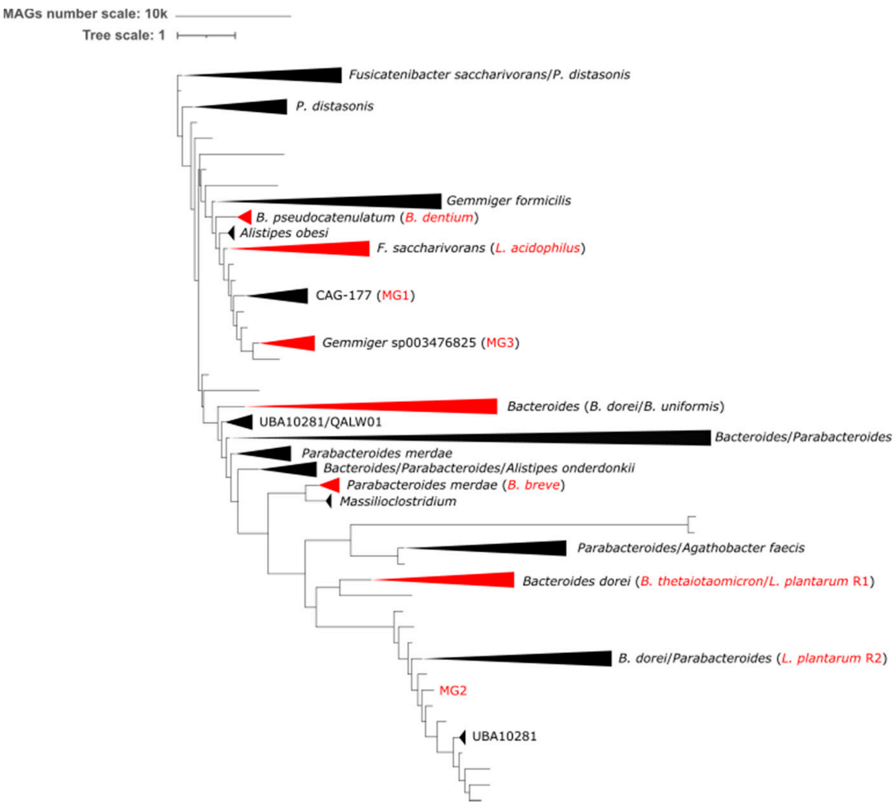

Supplementary Figure S5: Phylogenetic tree of rhamnosidase sequences. Most abundant species genomes are indicated besides the triangles. Colored in red are nodes containing the nine reference sequences (in brackets, for details see Table 1). Only nodes with an overall number of at least 500 genomes (including isolate genomes) were included as triangles, the lengths of the triangles represent the number of genomes as indicated by the scale.

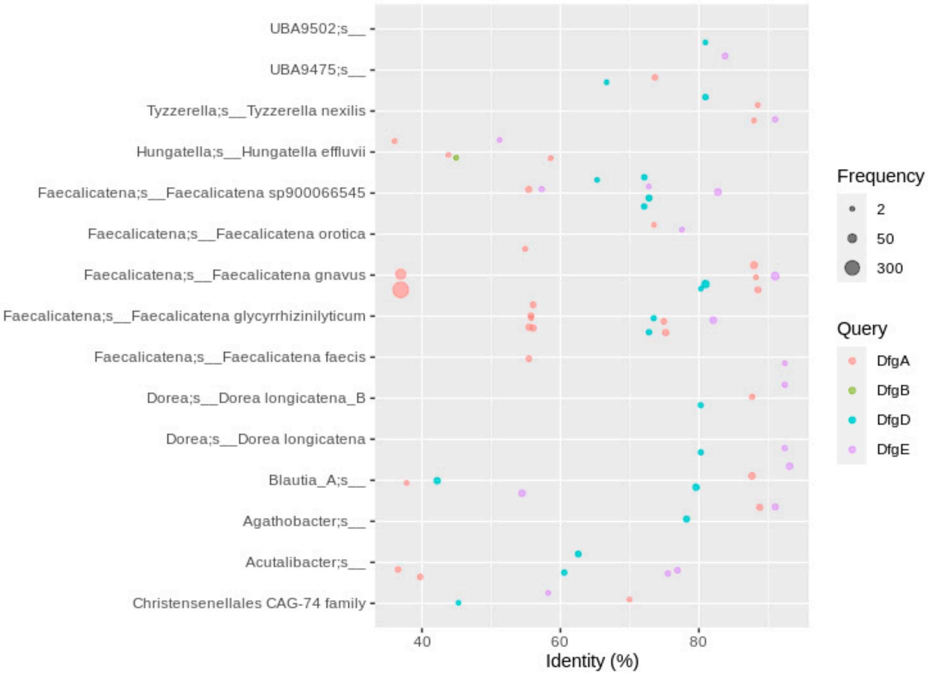

Supplementary Figure S6: Hits to the enzyme query sequences of the *C*-deglycosylation pathway of *E. cellulosolvens*. Filtered for the co-occurrence of at least three of the five enzymes and at least two identical/redundant sequences per bubble. There were no hits toDfgC.

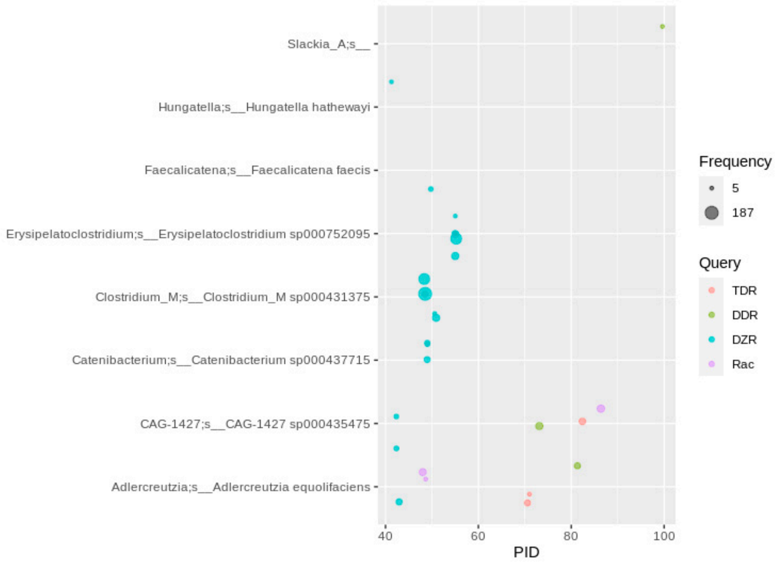

Supplementary Figure S7: Hits to daidzein-to-equol enzyme query sequences. Filtered was only for a PID of at least 40 and at least five occurrences of sequences per bubble, not for co-occurrences of the genes in one genome. Note the presence of a number of DZR hits at about 50 PID.

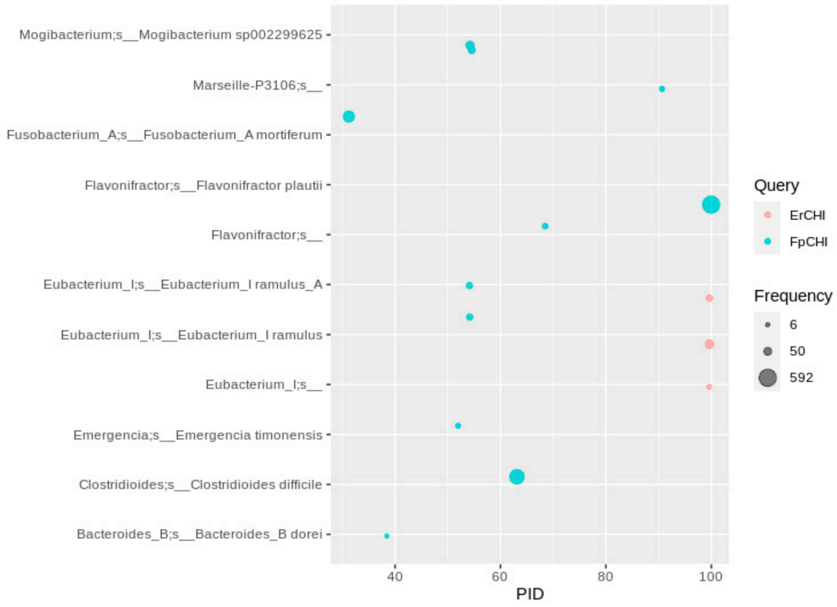

Supplementary Figure S8: CHI-like enzymes in human gut bacteria. Hits were filtered for at least five occurrences, so that each bubble represents a number of redundant sequences ranging from 6 to 592.

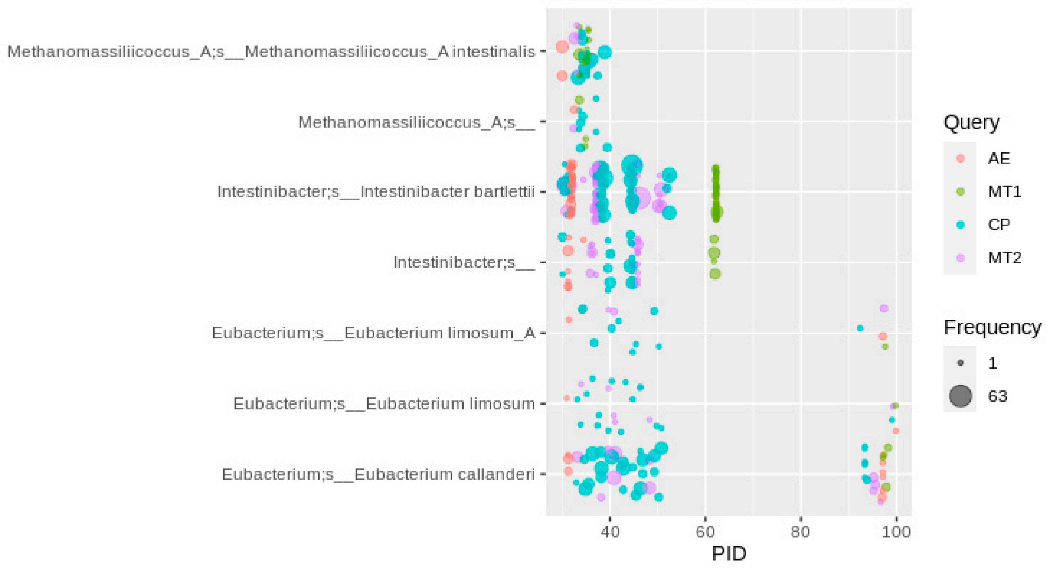

Supplementary Figure S9: Putative *O*-demethylase systems in human gut bacteria. Hits were filtered for the co-occurrence of all four genes encoding the enzymes required for *O*-demethylation (MT1, MT2, AE, CP) in one MAG.

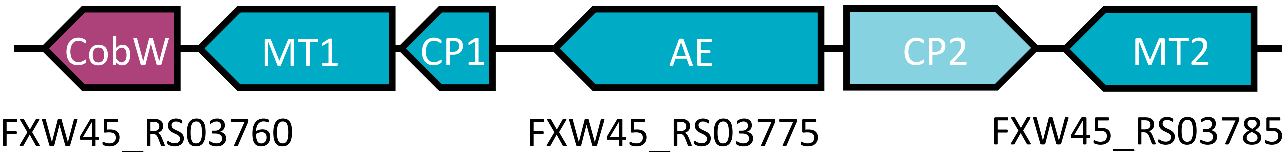

Supplementary Figure S10: *O*-Demethylase gene cluster of *Intestinibacter bartletti*. The representative MAG genome MGYG-HGUT-00062 (NCBI accession number NZ_CABIXZ010000001.1, GUT_GENOME000245) was used. Selected locus tags are given below the genes encoding the corresponding enzymes of *O*-demethylation (for abbreviations see Table 1). Accessory proteins colored violet. CP2 with a lower PID than CP1 to the *E. limosum* CP in light blue. NCBI protein accession numbers of main proteins: MT1: WP_147616460.1; CP1: WP_007286937.1; AE: WP_147616461.1; MT2 WP_007286933.1.

Supplementary Table S1: Distribution of BLAST hits (PID > 40) to flavonoid-modifying enzymes across all MAGs of species discussed in the text. For the number of MAGs with hits to flavonoid-modifying enzyme sequences, we counted the MAGs (genomes assigned to isolates in the UHGG metadata were excluded) carrying at least one BLAST hit to the corresponding enzyme sequence. Each genome was counted only once to avoid double counts when different hits to different queries were present.

Species	Number of MAGs with hits	Overall number of species MAGs
	***O*-Deglycosylation**	
Bifidobacterium adolescentis	2229	2404
Bifidobacterium pseudocatenulatum	1016	1063
Bifidobacterium infantis	1708	2717
Bacteroides thetaiotaomicron	489	553
Bacteroides ovatus	474	1027
Bacteroides cellulosilyticus	420	452
Bacteroides eggerthii	198	409
Faecalicatena gnavus	1155	1197
Gemmiger sp003476825	3184	3216
CAG-180 sp000434995	2226	2419
CAG-217 sp000436335	1124	1404
Blautia wexlerae	1871	2179
Eubacterium sp000434995	584	683
Faecalibacterium prausnitzii K	1243	1380
	**De-rhamnosylation**	
Bacteroides dorei	5246	5588
Bacteroides thetaiotaomicron	492	553
Bifidobacterium pseudocatenulatum	604	1063
Gemmiger sp003476825	2852	3216
Alistipes onderdonkii (unclassified Alistipes)	1800	1997
Parabacteroides distasonis	3782	4543
Paracacteroides merdae	1636	1723
	***C*-Deglycosylation**	
Faecalicatena gnavus (Ruminococcus gnavus)	82	1197
*Agathobacter faecis (*unclassified *Roseburia)*	804	2819
	**Dai-to-equol transformation**	
CAG1427 sp000435475	35	81
Adlercreutzia equolifaciens	33	271
	**Phy**	
Dialister succinatiphilus	318	345
Flavonifractor plautii	563	610
	**FLR**	
Fusobacterium mortiferum	165	270
Flavonifractor plautii	610	610
Clostridoides difficile	114	338
	**Fcr**	
Flavonifractor plautii	509	610
	**Chalcone isomerization/Taxifolin isomerization**	
Flavonifractor plautii	579	610
Clostridoides difficile	117	338
	***O*-Demethylation**	
Intestinibacter bartlettii	69	85

In brackets: Different taxonomy database entries (e.g. old/non-GTDB entries).

Supplementary Table S2: Potential flavonoid-modifying bacteria from a screening of the BRENDA enzyme database performed in a previous study [60].

Bacterium	Number of MAGs
Pantoea agglomerans	2
Acinetobacter johnsonii	1
Pseudomonas putida	1
Desulfitobacterium hafniense	1
Leuconostoc mesenteroides	5
Lactobacillus pentosus	14
Bacillus licheniformis	2
Bacillus subtilis	1
Bacillus cereus	4
